# Kinetical approach of release and antioxidant performances of ι-carrageenan-based films modified by sodium alginate and Ca^2+^ and incorporating sea fennel essential oil and its major components^☆^

**DOI:** 10.1016/j.fochx.2026.104155

**Published:** 2026-06-30

**Authors:** Lilou Avidos, Petra Pišonić, Mia Kurek, Frédéric Debeaufort, Nasreddine Benbettaieb

**Affiliations:** a University Burgundy Europe, IUT-Dijon, BioEngineering Dpt., 7 Blvd Docteur Petitjean, BP17867, 20178 Dijon Cedex, France; b University Burgundy Europe, Institut AgroDijon, INRAE, UMR PAM, Food Processing and Microbiology, 1 Esplanade Erasme, 21000 Dijon, France; c University of Zagreb, Faculty of Food Technology and Biotechnology, Pierottijeva, 10000 Zagreb, Croatia

**Keywords:** Bioactive films, Hydrocolloids, Release kinetics, Diffusivity, Partition, Antioxidant activity

## Abstract

Understanding whether antioxidant efficiency in active films is governed by release kinetics or intrinsic radical-scavenging reactivity remains insufficiently explored in hydrophilic biopolymer networks. This study investigates the coupling between release mechanisms and antioxidant reaction kinetics in ι-carrageenan-based films loaded with bioactive (chlorogenic acidor d-limoneneor sea fennel essential oil) to evaluate their release behavior and antioxidant activity. Release experiments were conducted in food simulant D1 (50% ethanol) over 48 h, while antioxidant activity (AA%) was monitored using the DPPHin the same simulant. Diffusion coefficients (D), kinetic rate constants, and times to reach 50% release (t_50% release_) or 50% antioxidant activity (t_AA50%_) were mathematically modeled.

The originality of this work lies in quantitatively and qualitatively correlation between release and antioxidant activity kinetics. Interpenetrated ι-carrageenan-alginate and Ca^2+^-crosslinked ι-carrageenan networks enable discrimination between release-controlled and antioxidant reaction-controlled. Chlorogenic acid exhibited the fastest release (≈27% after 48 h; D ≈ 32 × 10^−15^ m^2^.s^−1^) and radical-scavenging activity, reaching high AA% (46%), indicating a predominantly release-controlled system. In contrast, d-limonene and sea fennel essential oil displayed significantly lower diffusivity (from 4 to 17 times lower), lower and slower AA (AA% from 2 to 3 times lower; t_AA50%_ from 3 to 5 times higher) reflecting mass-transfer limitations and more complex reaction behavior within the matrix. Ca^2+^ crosslinking further modulated diffusivity and antioxidant performance by changing polymer network density. These findings provide mechanistic insights into how structural properties of hydrophilic polymer networks govern the balance between release and reactivity rates, offering a rational framework for designing active films with predictable and tunable antioxidant functionality.

## Highlights

• Chlorogenic acid has a faster release, stronger antioxidant activity (release-driven)
• d-limonene & sea fennel oil has slower diffusion, complex release profiles and weaker antioxidant activity
• Differences linked to whether antioxidant capacity is controlled by release rate or diffusion coefficient
• Adding Ca^2+^ and/or alginate allow to denser networks, slower release

## 1 Introduction

Fatty fish products are rich in ω-3 polyunsaturated fatty acids, which offer significant health benefits. This is particularly concerning, as these fatty acids play a vital role in the prevention of cardiovascular diseases (Gharby et al., 2025). However, their high unsaturated fat content makes them highly susceptible to lipid oxidation, leading to nutritional degradation and reduced shelf life (Wang et al., 2023). Lipid oxidation in food leads to the degradation of polyunsaturated fatty acids, generation of toxic aldehydes, formation of off-flavors and rancid odors, causing a significant loss in nutritional quality and/or making the product unsuitable for human consumption.

Various strategies have been employed to limit the rate and extent of lipid oxidation in foods and thereby extend the shelf life of oxygen-sensitive foods during their storage, including the direct addition of antioxidants to lipid-rich products. Antioxidants have thus been intentionally incorporated into products to prevent or slow down lipid oxidation during the processing and storage of fats, oils, and lipid-rich foods, and they have been a part of the food industry for over 60 years (Decker et al., 2010). However, this approach has several drawbacks, including the need for large quantities of antioxidants, the establishment of maximum allowed levels by food regulations in some cases, and the potential for high concentrations to act as pro-oxidants (Bohn et al., 2024).

Another approach involves developing packaging technologies such as vacuum or modified-atmosphere packaging and high-barrier materials, which can help limit oxygen transfer into packed foods (Czerwiński et al., 2021). However, some products, such as fresh fish, cannot be fully protected from oxidation through barrier packaging alone, and conventional systems often overlook quality changes during storage. For instance studies have often focused on processing techniques rather than the storage phase, with little knowledge of how temperature and packaging type influence the retention of bioactive compounds and antioxidant activity over time (Le et al., 2024). Similarly, for highly perishable foods coatings such as sodium alginate combined with calcium chloride and ascorbic acid can help maintain polyphenol content and antioxidant activity, but these approaches are still limited to short-term protection and do not systematically address controlled release of active compounds (Linh et al., 2024). These examples illustrate that traditional preservation and coating methods may only partially mitigate quality loss. Recent innovations in active packaging aim to overcome these limitations by incorporating natural antioxidant molecules into polymeric matrices or surface coatings, providing controlled release to maintain food quality. However, systematic studies correlating the kinetics of release and antioxidant activity in hydrophilic biopolymer networks remain limited, motivating the present work.

Sea fennel (Crithmum maritimum L.) is an edible halophyte plant recognized for its rich and complex chemical composition. It has gained growing research interest across various scientific disciplines, extending beyond its traditional applications in food and agriculture (Orhotohwo et al., 2024). Different forms of extracts from sea fennel flowers, stems, and leaves were previously prepared and studied, identifying limonene as the predominant compound in both essential oils and ethanolic extracts, with concentrations ranging from 57 to 74% and 0.7–8.1 mg/g of dry plant material, respectively. Furthermore, in ethanolic extracts, chlorogenic acid was a major component, ranging from 12 to 80 mg/g dry weight in the leaves and flowers of both cultivated and wild sea fennel populations (Nartea et al., 2023). Compared to Citrus essential oils, sea fennel essential oil has a milder aroma, which makes it more compatible with active packaging applications, allowing its use across a wider range of food products without imparting strong citrus odors. Additionally, the combination of its bioactive compounds may provide synergistic effects on antioxidant and antimicrobial activities (Nhi et al., 2022). These characteristics collectively justify the choice of sea fennel as a promising source of natural antioxidants (DL and chlorogenic acid) for incorporation into functional biopolymer films.

Thanks to its polyphenolic content and a variety of other bioactive compounds, sea fennel exhibits strong antioxidants and antimicrobial properties that are highly relevant to food packaging applications, contributing to improved food preservation and supporting the development of active packaging solutions (Kurek et al., 2024). Recent studies have shown that films and coatings incorporating sea fennel extracts can extend the shelf life of certain foods by up to 50% compared to untreated controls (Orhotohwo et al., 2024). As industry moves toward environmentally sustainable practices, sea fennel represents a promising natural source for the development of biodegradable and active films and coatings. Although its use in food packaging is still relatively new and remains underexplored by researchers, some works showed that it exhibits several desirable properties for edible packaging applications (Orhotohwo et al., 2024). Due to its natural origin and compatibility with marine-derived food systems, sea fennel essential oil may be particularly suitable for seafood applications like fresh fish fillets and further validation on these matrices is required to confirm its practical preservation efficacy.

In parallel, as consumer demand has shifted to safe and natural materials, seaweed has emerged as a valuable and cost-effective source of various biopolymers, including carrageenan, alginate, and agar, which can be used mainly as a responsive system for bioactive packaging (Kurek et al., 2024). Carrageenan, a polysaccharide derived from red seaweed (Rhodophyta), has attracted significant attention for its potential due to its excellent film-forming properties (Kokkuvayil Ramadas et al., 2024). Carrageenan consists of water-soluble hydrocolloids with a linear chain of sulfated galactans. Its structure is characterized by the presence of β-(1,3) sulfated D-galactose and α-(1,4)-3,6-anhydro-D-galactose residues (Prasetyaningrum et al., 2021).

Carrageenans are classified into κ-, ι-, and λ-types according to the number and position of sulfate groups. κ-Carrageenan possesses stronger gel-forming ability due to its lower sulfate ester content (one sulfate group per disaccharide unit), forming rigid and brittle gels or films that typically require the incorporation of plasticizers to improve flexibility (Monteiro et al., 2025). In contrast, ι-carrageenan contains two sulfate groups and was selected in this study for its ability to form softer and more elastic gels, particularly in the presence of specific cations that enhance its functional properties (Thrimawithana et al., 2010). The higher sulfate content of ι-carrageenan promotes greater network flexibility and improved compatibility with hydrophilic active compounds, which may facilitate more controlled release behavior. Moreover, ι-carrageenan-based films generally require lower plasticizer content and exhibit favorable water resistance and retention properties (Paula et al., 2015). These characteristics are particularly relevant for active packaging applications, where film flexibility and controlled release of incorporated compounds are critical. Indeed, Yang and Sun reported that carrageenan can form transparent films with excellent mechanical and physical properties (Yang & Sun, 2025). However pure carrageenan has some limitations when used for film development, including high hydrophilicity, poor water barrier properties, poor O_2_ barrier properties at higher humidity. The functional and physical properties of carrageenan can be improved by combining it with other biopolymers, nanofillers, bioactive ingredients, and crosslinking agents (Ahmed et al., 2024; Kokkuvayil Ramadas et al., 2024; Rammak et al., 2021).Alginate, a natural polysaccharide extracted from brown algae (Laminariaceae and Fucaceae),shows great potential for development as a bio-edible film, owing to its versatility, non-toxicity, biocompatibility, gel-forming ability, and biodegradability (Prasetyaningrum et al., 2021) and can for interpenetrated network with carrageenans. The chemical structure of alginate consists of *β*-(1,4)-linked *D*-mannuronic acid units (M) and *L*-guluronic acid units (G) (Li et al., 2024).

Numerous studies have investigated the blending of carrageenan with other polysaccharides to enhance the mechanical, barrier, and functional properties of the resulting films (Bhatia et al., 2024; Cheng et al., 2022; Kokkuvayil Ramadas et al., 2024). Due to their mild ionotropic gelation in the presence of divalent cations (e.g., Zn^2+^, Ca^2+^), these hydrocolloids are widely explored for encapsulation and controlled delivery applications, making them attractive candidates for active packaging (Zhang, Zhang, et al., 2024; Zhang, Zuo, et al., 2024). Interpenetrating polymer network organization may contribute to more controlled release of incorporated active compounds during storage. Indeed, both carrageenan and alginate are capable of interacting with di- and trivalent cations, especially calcium ions, leading to ionic crosslinking and enhanced network cohesion. Despite these advantages, the influence of divalent cation crosslinking on the controlled release behavior of active compounds from hydrocolloids-based films remains insufficiently understood.

Alginate and carrageenan-based films incorporating essential oils have demonstrated physicochemical, mechanical, and antimicrobial and antioxidant properties depending on environmental factors such as pH and relative humidity. These effects on film stability and functional properties have been reported in previous studies (Prasetyaningrum et al., 2021) and (Barbut & Harper, 2019) highlighting the importance of formulation and conditioning for specific applications.

Sea fennel contains chlorogenic acid and d-limonene as their major bioactive constituents. Chlorogenic acid, a phenolic compound, exhibits strong antioxidant activity due to its molecular structure, which includes multiple hydroxyl groups and a carboxyl group capable of donating hydrogen atoms and stabilizing free radicals through resonance. This structural feature confers high radical-scavenging efficiency. Recent studies have incorporated chlorogenic acid into bio-based films such as cellulose- and chitosan-based matrices, demonstrating enhanced antibacterial and antioxidant performance and improved food shelf-life (Dai et al., 2024). Although the antioxidant capacity of sea fennel extract and its principal components have been previously evaluated, and their release from polymeric films into food simulants has been reported, the direct and dynamic relationship between release kinetics and antioxidant reaction kinetics has not been systematically investigated. Elucidating this coupling represents a key novelty and scientific contribution of the present study.

The kinetics of active compound release can critically influence antioxidant efficacy. If release is too slow, the antioxidant concentration may remain below the effective threshold; if too fast, an initial burst effect may rapidly deplete the compound, shortening the protection window. Moreover, uncoordinated release to antioxidant activity can render antioxidants ineffective, either by being delivered too early or too late. Despite its importance, current literature lacks systematic kinetic studies that simultaneously correlate antioxidant activity with controlled release in such systems. Understanding the coupling between release kinetics and antioxidant reactivity, in relation to film structural properties, is therefore still missing. Addressing this gap is essential for designing active films with predictable and sustained functional performance. The originality of the present study lies in evaluating how release can act as a limiting step or not for antioxidant activity. Specifically, we investigated the release of sea fennel hydrodistillated oil, d-limonene, and chlorogenic acid from carrageenan- and alginate-based films into aqueous food simulants, while simultaneously monitoring antioxidant activity using the DPPH assay. The target lies with fresh foods/fillets with short shelf-life, requiring quite fast release and fast antioxidant activity after packaging. Kinetic parameters (rates, diffusivity, partition) were, thus, determined through mathematical modeling to elucidate underlying mechanisms, with D-limonene and chlorogenic acid serving as tracers for the more complex sea fennel oil, while the effect of Ca^2+^ crosslinking on carrageenan-based network behavior was also examined.

## 2 Materials and methods

### 2.1 Materials

Commercial grade of iota-carrageenan (Car) (Louis François, E407 gelling agent, MW = 1–1.2 kDa (10.10^5^ - 12.10^5^ g.mol^−1^), viscosity, Croissy Beaubourg, France) and sodium alginate (Alg) with a M/G ratio of about 0.6 (Fisher Scientific, high viscosity, Waltham, US) were used as film-forming matrices. Calcium chloride (CaCl_2_, MW = 110.98 g.mol^−1^, anhydrous, granular, ≤ 7.0 mm, purity 95–97%, Prolabo, Croissy Beaubourg, France) was used as crosslinker, and anhydrous glycerol (Fluka Chemical, 98% purity, Seelze, Germany) as a film plasticizer. Ethanol (Fisher Scientific, analytic reagent grade: 96° (*v*/v)) was used to prepare the food simulant D1 (ethanol-water: 50–50% (v/v)) for the release test.

Sea fennel (SF) essential oil was obtained by hydrodistillation (purity ˃ 98% OPG, Grgo Lučić, Zastrašišće, Croatia), chlorogenic acid (CA) was externally purchased (Tokyo Chemical Industry (TCI), MW = 354.31 g.mol^−1^, purity ˃ 98%, Tokyo, Japan) and d-Limonene (DL) was externally sourced (analytic reagent grade MW = 136.23 g.mol^−1^, density = 0.842 g.mL^−1^ at 20 °C, Fluka, Saint Louis, US) and was used as active ingredients. The chemical structure and the physicochemical characteristics of antioxidant tracers of sea fennel oil are given in Table 1. The composition of the essential oil of sea fennel depends on location, season, part of the plant and process parameters of the hydrodistillation. The major compounds were listed from literature data (Generalić Mekinić et al., 2024; Politeo et al., 2023; Slišković et al., 2025; Šunić et al., 2024). Indeed, from these authors, sea fennel essential oil from creation contains mainly d-Limonene (from 39 to 79%), Sabinene (0.8 to 42%), γ-Terpinene (11–14%), β-Ocymene (2 to 4.4%), p-Cymene (1 to 2.5%), Terpinene-4-ol (0.6 to 19.2%), Dillapiol (0.7 to 3.3%) and Carvacrol (0.7 to 3.3%).

**Table 1:** Physical-chemical parameters of tracers able to mimic sea fennel (*data from**www.ChemSpider.com**)*.

Structure and physical-chemical parameters	Chlorogenic acid (CA)	d-Limonene (DL)
**Chemical structure**		
Chemical formula	C_16_H_18_O_9_	C_10_H_16_
Chemical name	trans-5-O-caféoyl-D-quinate	1-methyl-4-(1-methylethenyl)-cyclohexene
CAS	327–97-9	5989-27-5
molecular weight (g.mol^−1^)	354.31	136.24
molar volume (cm^3^.mol^−1^)	276.8	160.9
density (ρ) (g.cm^−3^)	1.28	0.86
melting point MP (°C)	207	−74.35
boiling point BP (°C)	665	176
solubility in water at 20 °C (g.L^−1^)	40	0.0075 to 0.0138^b^
pKa in water or aqueous solution	3.5–3.6 and 8.3–8.6 a	2.92, 4.28 and 5.21 c
Log P (water/octanol partition)	0.45	4.5

a (Timberlake, 1959).

b (Massaldi & King, 1973; Miller & Hawthorne, 2000).

c (National Institute of Health, 2026).

### 2.2 Active film preparation

Carrageenan (Car) or sodium alginate (Alg) powder were separately dispersed (at 3% *w*/*v*) in distilled water for 30 min using a helices-pale stirrer (RW16 basic- IKA-WERKE, Staufen, Germany) at 8000 rpm and at 65 °C using water bath to prepare basic film forming solutions (FFS). After proper homogenization, glycerol (15%, *w*/w dry matter of biopolymer) was added to each FFS, with continuous stirring (8000 rpm) for further 5 min. A mixed carrageenan‑sodium alginate (Car-Alg) FFS was prepared by mixing both Car and Alg dispersions at equal volume (50–50, *v*/v), maintaining the same stirring and temperature as for basic FFS. Carrageenan and sodium alginate have complementary properties: carrageenan provides good film-forming ability and mechanical strength, while sodium alginate improves flexibility and barrier properties. A 50:50 (*w*/w) ratio was chosen because it balances these properties. This ratio is also supported by previous studies showing optimal film integrity and homogeneity at a 1:1 (w/w) ratio (Paula et al., 2015). The pH of each solution was adjusted to 6 using lactic acid solution (at 10%, v/v). d-Limonene (DL) and sea fennel oil (SF) were added separately to Car and Car-Alg FFS at a concentration of 5% (w/w of dry matter of biopolymer) under stirring. Chlorogenic acid (CA) was added to Car at a concentration of 1% (w/w of dry matter of biopolymer). These amounts were selected to respect the relative proportion of each in the sea fennel as chlorogenic acid is one of the major phenolic compounds in sea fennel ethanolic extracts whereas d-limonene is one of the predominant constituents of sea fennel essential oil and may represent more than 80% of the volatile fraction. This approach allows for a formulation strategy that remains representative of the original sea fennel composition. In addition they were also selected based on literature (Jacobs et al., 2023) and preliminary tests as optimal amount that ensures significant antioxidant and bioactive activity without compromising the mechanical integrity and transparency. An aliquot of 40 mL of each FFS was then poured into square plastic Petri dishes (12.2*12.2 cm^2^). CaCl_2_ (used as crosslinker) was solubilized in water at a concentration of 20 g/L and then around 1 mL was sprayed onto freshly cast FFS to reach a ratio of about 1.6% CaCl_2_ to the weight of dry biopolymer. CaCl_2_ was added at 1.6% (*w*/w) relative to the dry biopolymer content based on the stoichiometric interaction between Ca^2+^ ions and sulfate groups (–SO_3_^−^) of ι-carrageenan. Each repeating unit of ι-carrageenan contains two sulfate groups, and one Ca^2+^ ion can electrostatically interact with these sulfate groups, forming ionic crosslinks within the polymer network. The selected CaCl_2_ concentration was calculated to provide sufficient Ca^2+^ to interact with the available sulfate groups, promoting network reinforcement while avoiding excessive crosslinking that could adversely affect film flexibility and controlled release properties. The spraying method ensured uniform CaCl_2_ distribution on the dried film, as direct addition to the solution caused rapid gelation and prevented proper casting. These conditions were maintained consistently across all experiments to allow reliable comparative evaluation of release kinetics and antioxidant activity.

The FFS solvent was removed (for 18 to 24 h) by drying at about 35% using a fan heater located at 25 cm from Petri dishes. After drying, the films were peeled off from the surface and stored between aluminum foils at around 25 °C before analysis. All films formulations have been coded as described in Table 2.

**Table 2:** Film sample code and description.

Film sample code	Description
Car	Carrageenan films
Car-CA	Carrageenan film incorporated chlorogenic acid at 1% (*w*/w)
Car-DL	Carrageenan film incorporated d-Limonene at 5% (w/w)
Car-SF	Carrageenan film incorporated Sea fennel at 5% (w/w)
Car-CaCl_2_	Carrageenan films crosslinked by CaCl_2_
Car-CaCl_2_-SF	Carrageenan films incorporated Sea fennel at 5% (w/w) and crosslinked by CaCl_2_
Car-Alg	Carrageenan‑sodium alginate film (50–50, w/w)
Car-Alg-SF	Carrageenan‑sodium alginate film (50–50, w/w) incorporated Sea fennel at 5% (w/w)

### 2.3 Film thickness measurement

The film thickness was measured at least five different positions using a Micrometer Thickness Gauge with a resolution of 0.001 mm (F50, Hans Schmidt & Co GmbH; D-84478, Waldkraiburg, Germany). The average value was used for further calculations and the standard deviation considered for relative errors.

### 2.4 Antioxidant efficacy measured by DPPH method

Antioxidant activity (AA, %) of the films was assessed using the stable free radical 2,2-diphenyl-1-picrylhydrazyl (DPPH^•^). In this study, AA refers specifically to radical scavenging activity (RSA) toward DPPH radicals and does not encompass other antioxidant mechanisms. Film samples (6 cm^2^, regardless of formulation) were placed in a glass vial containing 10 mL of a DPPH solution (50 mg/L) prepared in ethanol 50%. The chosen film surface-to-solution volume ratio was consistent with the ratio used for release kinetics (60 cm^2^ of film per 100 mL of liquid food simulant) in accordance with European Regulation EC/10/2011 (European Commission EC/10/2011, 2011) on migration testing. Importantly, the use of 50% ethanol aqueous solution aligns with the release studies conducted in food simulant D1 (dedicated to fatty foods), enabling meaningful comparison between release and antioxidant activity kinetics. Complementary assays, such as ABTS, ORAC, or FRAP, could provide additional insights, particularly for hydrophobic compounds like d-limonene and essential oils. However, these assays are generally conducted in purely aqueous or different solvent systems, making them less suitable for direct comparison with the D1 release medium (Pham et al., 2024). The chemical stability of CA and major SF components during the 48 h release in 50% ethanol was carefully considered, with experiments conducted under controlled light and temperature to minimize degradation. Minor oxidation or isomerization, if any, is expected to have negligible impact on comparative release trends and measured antioxidant activity.

To prevent a light-induced degradation of DPPH^•^, the glass vials were covered with aluminum foil. The reaction kinetics was monitored by measuring the disappearance of the DPPH^•^ radical through absorbance readings at 517 nm using a Jenway 6305 UV–Visible spectrophotometer (Thermo Fisher Scientific, Waltham, MA, USA). During the kinetic measurements, 2 mL of the reaction solution was sampled for absorbance reading and subsequently returned to the glass vial to maintain a constant volume. Samples were kept in sealed vials and stirred until the end-time of experiment (time corresponding to equilibrium for the release test).

The antioxidant activity (AA, %) was expressed as the percentage of DPPH^•^ inhibition and calculated using the following equation (Eq. (1)):

(1) $$\mathrm{AA}\left(t\right)=\frac{A_{\mathrm{blank}\;\left(t\right)}-A_{\mathrm{sample}\;\left(t\right)}}{A_{\mathrm{blank}\;\left(t\right)}}\times 100$$

where $A_{\mathrm{blank}\;\left(t\right)}$ represents the absorbance of the DPPH solution without the film at time t, and $A_{\mathrm{sample}\;\left(t\right)}$ represents the absorbance of the DPPH solution containing the film sample at the same time.

During antioxidant activity kinetics, the optical density (OD) of the DPPH solution alone (blank, without active compounds or films) was also monitored simultaneously with the OD of samples containing active films or pure compounds. This approach allowed correction for potential DPPH self-decay during the assay period. At each time point, the absorbance of the sample was substracted from the absorbance of the corresponding blank DPPH solution to account for any minor changes in radical stability over time. This procedure ensured that the calculated antioxidant activity reflected only the radical scavenging effect of the tested compounds.

Additionally, to compare the antioxidants activity of pure active molecules, 0.5 mg of CA and 2.5 mg, of DL or SF were weighed and directly incorporated into 10 mL of DPPH medium. These amounts of pure antioxidants (mg) were calculated to be very close to those contained in the 6 cm^2^ of films. Three repetitions were done for each formulation. This test was recorded overtime until 48 h and the antioxidant activity (AA, %) at equilibrium (end of the kinetic process) was calculated for all formulation (pure active molecules, films with and without active molecules). The time to reach a half activity (t_AA50%_) to the maximum value at equilibrium was also displayed. Due to absence of the lag phase in the AA (%) during the time, the kinetic rate constant (%.min^−1^) was determined as the slope of linear part of the curve (AA vs. time until t_AA50%_) and discussed as the rate of DPPH free radical scavenging.

To verify whether calcium ions could directly interact with DPPH radicals, a control experiment was performed using Ca^2+^ solutions at the same concentration present in the crosslinked films (1.6% of dry polymers). Specifically, 0.8 mg of CaCl_2_ was dissolved in 10 mL of DPPH medium. The solution was incubated under the same condition as the film tests, and the antioxidant activity (AA, %) was recorded over 48 h and calculated at the same manner used for pure compounds and active films. This experiment was done in triplicate and allowed distinguishing between matrix-mediated effects on DPPH scavenging and any potential direct contribution from Ca^2+^ ions.

### 2.5 Release kinetics of antioxidants from active film to food simulant

The kinetic release of active molecules from carrageenan films and carrageenan‑sodium alginate films was evaluated through total double-sided immersion in a food simulant, following European Regulation 10/2011.

Food simulant D1 (50% *v*/v ethanol) was selected in accordance with European Commission Regulation No. 10/2011 (European Commission EC/10/2011, 2011) for food contact materials intended for fatty and high-moisture foods. The 50% ethanol system provides intermediate polarity, enabling the dissolution and comparative evaluation of both hydrophilic compounds (e.g., chlorogenic acid) and moderately hydrophobic compounds (e.g., d-limonene and sea fennel essential oil) under standardized migration conditions. The developed films are intended for application in fresh fish fillets, which are characterized by high water activity, the presence of oxidizable lipids, and a short refrigerated shelf life (approximately 3–5 days). Therefore, the use of D1 simulant was also considered appropriate to assess antioxidant release behavior under conditions relevant to the targeted food system.

For this test, film samples (60 cm^2^) were immersed in glass vials containing 100 mL of D1 simulant stirred at 630 rpm to minimize the boundary layer effects at the film interface for non-viscous liquids and for films of similar viscosity, dimensions, and density (Jacobs et al., 2023). The selected film surface-to-simulant volume ratio (60 cm^2^/100 mL) followed the standard test protocol for measuring migration (European Commission EC/10/2011, 2011; European Commission EU/2016/1416, 2016). All kinetic experiments were performed at 25 °C. While this provides a controlled baseline, potential variations in storage temperature may affect release and antioxidant activity, as discussed later in R&D part. Indeed, the chemical stability of active compounds (chlorogenic acid, d-limonene, and sea fennel essential oil) was considered as stable during the 48-h release experiments. To minimize degradation, isomerization, or oxidation, experiments were conducted at 25 °C and protected from direct light.

Approximately 1.5 mL of the food simulant was periodically sampled until equilibrium was reached, and UV–Vis scans from 200 to 400 nm were performed using a UV–Vis spectrophotometer (Thermo Scientific GENESY 50 UV–Visible Light Spectrophotometer, France). The absorbance (OD) was recorded for each active molecule at its corresponding maximum wavelength (λ_max_).

The λ_max_ values at 208 nm for both d-Limonene and Sea Fennel, and at 324 nm for chlorogenic acid were previously determined from the complete UV spectra of the pure active molecules in D1 simulant at a concentration of 10 mg/100 mL. The release of sea fennel essential oil (SF) was monitored using UV absorbance at 208 nm as a global signal representative of the overall extract concentration. Given the multicomponent nature of essential oils, this method provides an apparent quantification of the total released fraction rather than the concentration of individual constituents. Since SF is a multicomponent extract, the calculated D and K values are considered apparent formulation-dependent parameters describing the global release behavior rather than the diffusion of individual compounds. The released active compounds were quantified using calibration curves prepared with standard concentrations of 0, 1, 2.5, 5, 10, 15, and 20 mg of pure active molecules in 100 mL of food simulant, following Beer-Lambert's law with significant linearity (R^2^ > 0.92). All experiments were performed in triplicate.

During this test, the UV spectra of control films (Car, Car-CaCl_2_ and Car-Alg) were also recorded in triplicate over time. The Langmuir equation (Eq. (2)) was applied to the release kinetic (x = time, y = mean of experimental OD (optical density) over time for the three repetition) for each control film (Car, Car-CaCl_2_ and Car-Alg).

(2) $$\mathrm{yfit}\left(t\right)=\frac{K.N_{0}.t}{1+k.t}$$

The Langmuir parameters (*K* and *N*_*0*_) were then determined using Matlab/Simulink software environment (Matlab 8.5–2015) and applied to mean of experimental values (OD-experimental from the kinetics) for each control film. These parameters were then used to discriminate the contribution of the Car, Car-CaCl_2_ or Car-Alg in the OD of active films.

Then the corrected OD values from the previously modelized Langmuir-kinetic y-fit (OD-fit) of each control film were substracted from the experimental OD values of the corresponding active films at each corresponding time point. This approach ensures that only the contribution from the release of active compounds is considered.

To find out the mechanism of active compounds release, first 60% release data were fitted with the Korsmeyerñ Peppas model according to Eq. (3).

(3) $$\frac{C_{S,t}}{C_{S,\infty }}=k.t^{n}$$

where $\frac{C_{S,t}}{C_{S,\infty }}$ is a fraction of active molecules released at time t, k is the release rate constant, and n is the release exponent. The n value is used to characterize different releases for plane shaped matrices. In this model, the value of n characterizes the release mechanism of active molecules. From Korsmeyerñ Peppas model *n* < 0.45 corresponds to a Fickian diffusion mechanism (Korsmeyer et al., 1983). In our case all n were lower than 0.45, supporting the assumption that mass transport is primarily governed by Fickian diffusion rather than polymer relaxation, erosion, or time/concentration-dependent effects. The diffusion of the antioxidant, from active film into food simulant was therefore determined from the Fick's second law. The analytical solution of the second Fick's law (Eq. (4)) in the case of transient state and for a limited volume of film immersed in a limited volume of liquid, was proposed by (Crank, 1975). The analytical solution supposes some preliminary assumptions: initial conditions and boundary conditions:

Preliminary assumptions:

➢ The liquid medium is perfectly stirred; therefore, no boundary layer affects mass transfer. The antioxidant leaves the film at the same rate as it enters the simulant medium, meaning that liquid-side mass transfer resistance is negligible;
➢ Diffusion occurs unidirectionally, from the center of the film toward its surface;
➢ The diffusivity of the active compound is constant, i.e., it is neither concentration-dependent nor time-dependent.

Initial conditions (*t* = 0):

At time zero (t = 0), the concentration of the antioxidant in the film (C_F_) is uniform and equal to the initial concentration (C_0_) incorporated during the film-forming process. The concentration of the antioxidant in the food simulant (C_S_) is assumed to be zero.

Boundary conditions (*t* > 0):

For times greater than zero, the rate at which the active compound leaves the film is equal to the rate at which it enters the surrounding simulant solution. The transport kinetics of the active compound from a plane sheet of thickness 2 L (with both faces exposed to the simulant) were described by (Crank, 1975) according to the following equations:

(4) $$\frac{C_{t,S}}{C_{\infty ,S}}=1-\sum_{n=1}^{\infty }\frac{2\alpha \left(1+\alpha \right)}{1+\alpha +\alpha^{2}q_{n}^{2}}\exp\left(-\frac{Dq_{n}^{2}t}{L^{2}}\right)$$

where:

(5) $$\alpha =\frac{V_{S}}{\left(K_{F/S}\times V_{F}\right)}$$

D is the effective diffusion coefficient (m^2^.s^−1^), V_F_ is the film volume (m^3^) and $K_{F/S}$ is the partition coefficient of the active compound between the film and the simulant:

(6) $$K_{F/S}=\frac{C_{\infty ,F}}{C_{\infty ,S}}$$

$C_{\infty ,F}\;$is the concentration (mg/L) of the antioxidant in the film and $C_{\infty ,S}$ that in the food simulant at equilibrium (at infinite time).

Finally, the $q_{n}$ are the non-zero positive roots of Eq. (7).

(7) $$\tan\left(q_{n}\right)=-\alpha q_{n}$$

This model (Eq. (4)) was previously adapted and applied to the experimental release kinetics (up to equilibrium) (Benbettaïeb et al., 2020). The model parameters were estimated by minimizing the sum of squared differences between the experimental and predicted values using the Levenberg–Marquardt nonlinear regression algorithm. The diffusion coefficient (D) was treated as the adjustable parameter, with the number of terms in the series solution (n) set within the range of 0–1000 (Jacobs et al., 2023). Modeling was performed using Matlab/Simulink software environment (Matlab 8.5–2015). Model calculation were triplicate analyses. From each calculation, statistical parameters such as R^2^ and standard errors of the fitted coefficients (RMSE, mg/L) were calculated to ensure reliable estimation of the kinetic parameters (Khan et al., 2022)

(8) $$\mathrm{RMSE}=\sqrt{\frac{\sum_{i=1}^{N}{\left({\hat{\gamma }}_{\dot{i}}-y_{i}\right)}^{2}}{N}}$$

where, ${\hat{\gamma }}_{\dot{i}}$ $\text{and}\;y_{i}\;$were respectively predicted and experimental residual values while N is the number of experiment point.

Diffusivity values correspond to apparent/effective diffusion coefficients (D) rather than intrinsic material constants. D apparent reflects not only the inherent diffusivity of the active compound in the solvent but also the influence of the polymer matrix structure (network density, mesh size) and interactions between the molecules and the polymer network (molecular volume, polarity, and solubility). Therefore, these values provide a practical measure of mass transport under the experimental conditions, rather than a purely intrinsic material property.

### 2.6 Statistical methods

Statistical analysis was conducted using SPSS 13.0 software (Stat-Packets Statistical Analysis Software, SPSS Inc., Chicago, USA). A one-way analysis of variance (ANOVA) was performed to identify significant differences through multiple mean comparisons. Prior to ANOVA, assumptions of normality and homogeneity of variance were evaluated using the Shapiro–Wilk test and Levene's test, respectively. When ANOVA indicated significant differences, means were compared using the least significant difference (LSD) post hoc test. Statistical significance was set at *p* < 0.05.

For comparisons between two groups a Student's *t*-test was used to assess differences between the means at a 95% confidence level. The statistical approach adopted in this study is consistent with previously reported methodologies for evaluating antioxidant kinetics and release behavior (Jacobs et al., 2023).

## 3 Results and discussion

The antioxidant efficiency of active films is strongly related to both the intrinsic antioxidant activity of the active molecules and to their ability to be released. Indeed, the release parameters such as the apparent diffusivity of the active molecules in the film and their partition between the film and food simulant (link to their solubility on both phases) are film-structure dependent. Consequently, the presence of a bivalent cation that could act as a crosslinker in the biopolymer network could affect the diffusivity of the active molecule. Understanding the relationship between the kinetic of bioactivity (antioxidant activity using DPPH assay) of active compounds and their kinetics of release from biobased films into D1 food simulants is necessary for optimized responsive packaging. Car-CaCl_2_ and Car-Alg films were included as negative controls. While these films contain no added active compounds (SF, CA, or DL), carrageenan and CaCl_2_ can exhibit low-level intrinsic antioxidant activity. Since no actives are present, no release data were measured or reported for these controls.

### 3.1 The antioxidant activity of pure active ingredients and non-functionalized films

**Fig. 1:**
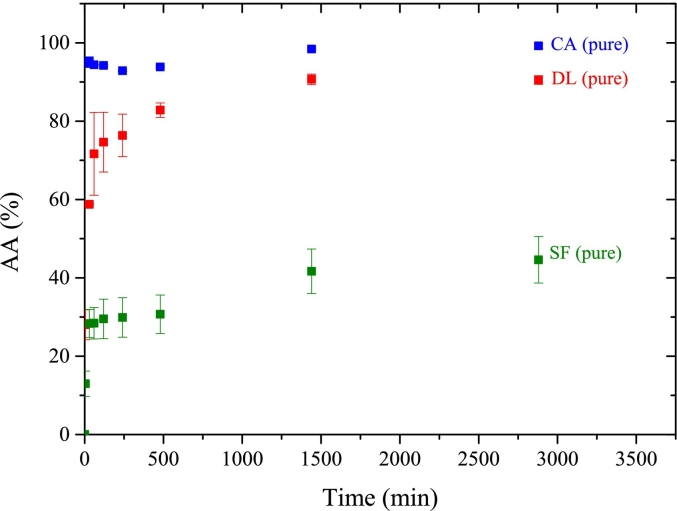
Kinetic of antioxidant activity of pure chlorogenic acid (CA), d-Limonene (DL) and sea fennel extract (SF) using DPPH assay in ethanol 50%.

Fig. 1 shows the antioxidant activity kinetics of pure active molecules (CA, DL and SF). The stability of the DPPH solution according the kinetic experiments time was first verified, and no significant change in optical density (OD) was observed, these control data have been included in the Supplementary Information (Fig. S1.a).

The maximum AA of chlorogenic acid (CA) (99.21 ± 0.58%) at equilibrium was significantly higher than that of DL (90.67 ± 1.11%) and SF (44.61 ± 5.94%) (Fig. 1). This ranking reflects the observed DPPH assay resulted from the experimental conditions and may not fully represent intrinsic antioxidant capacity, particularly for hydrophobic compounds due to relative solubility of the bioactive. Chlorogenic acid requires only 2.62 min to reach the AA_50%_ compared to 16.76 and 20.83 min for DL and SF, respectively (Table 3). In addition, the AA kinetic rate of CA (18.93 ± 0.09%.min^−1^) was from 3 to 7 times faster than that of DL (5.60 ± 0.76%.min^−1^) and that of SF (2.59 ± 0.63%.min^−1^). It was found that CA, even at only 1% (*w*/*v*), reacted faster, and led to a total quenching of the free DPPH^•^ radical compared to other compounds at higher concentrations (5% w/v). In general, the chemical structure of active compounds is a key determinant of their radical scavenging activity, and this is referred to as structure-activity relationships. This relationships of essential oil and flavonoids are generally more complicated than those of phenolic acids due to their relatively more complex composition. Chlorogenic acid is an ester of caffeic acid and quinic acid that contains 5 hydroxyl group responsible for its stronger radical scavenging acid capacity. The degree of hydroxylation as well as the position of the hydroxyl groups increased the radical scavenging capacity. The presence of a para and meta-OH on the caffeic acid ester ring acts as a hydrogen-bond donor group or electron-donating group enhancing the AA of CA. The strong AA of CA is due to the unpaired electron delocalization, able to quench the free radicals through hydrogen atoms, and to a single electron transfer mechanism (Mingsan & Liling, 2020).

**Table 3:** Antioxidant activity and release kinetics parameters of different formulations (pure active molecules, control films and active films).

Formulation	Antioxidant activity parameters			Release kinetics parameters			
	AA (%) at equilibrium	t_AA50%_ (min)	Kinetic rate (% . min^−1^)*	% of release at equilibrium ((C_t_/C_0_)_∞_)	t_50% release_ (min)	Diffusion coefficient (10^−15^ m^2^/s)**	Partition coefficient $K_{F/S}=\frac{C_{\infty ,F}}{C_{\infty ,S}}$
CA-pure	99.21 ± 0.58 ^a^	2.62 ± 0.04 ^h^	18.93 ± 0.09 ^a^				
DL-pure	90.67 ± 1.11 ^b^	16.76 ± 4.78 ^g,f^	5.60 ± 0.76 ^b^				
SF-pure	44.61 ± 5.94 ^c^	20.83 ± 1.3 ^f^	2.59 ± 0.63 ^c^				
Car	12.79 ± 4.13 ^g^	306 ± 176 ^d,c^	0.087 ± 0.012 ^g^				
Car-CA	46.31 ± 3.71 ^c^	22 ± 2.64 ^f^	0.55 ± 0.06 ^d^	27.44 ± 1.27 ^a^	50 ± 10 ^e^	32 ± 3.46 ^a^	735 ± 42.8 ^e^
Car-DL	22.31 ± 5.42 ^e,f^	70 ± 14.14 ^e^	0.14 ± 0.028 ^e^	21.95 ± 3.5 ^b^	87.5 ± 3.53 ^d^	7.50 ± 0.28 ^b^	1093 ± 76.36 ^d^
Car-SF	15.81 ± 1.93 ^g,e^	100 ± 48 ^d^	0.091 ± 0.054 ^g^	10.13 ± 0.38 ^c^	185 ± 21.21 ^c^	1.83 ± 0.6 ^c^	2406 ± 82 ^c^
Car-CaCl_2_	26.20 ± 2.35 ^e,f^	490 ± 14.14 ^b^	0.06 ± 0.0007 ^e^				
Car-CaCl_2_-SF	34.85 ± 1.64 ^d^	210 ± 10 ^c^	0.07 ± 0.004 ^h^	2.82 ± 0.37 ^d^	370 ± 34.64 ^b^	0.075 ± 0.005 ^d^	9550.6 ± 1371 ^b^
Car-Alg	14.99 ± 0.77 ^g^	750 ± 70.7 ^a^	0.035 ± 0.022 ^i^				
Car-Alg-SF	28.03 ± 3.42 ^e^	280 ± 105 ^c^	0.11 ± 0.007 ^f^	0.33 ± 0.024 ^e^	456.6 ± 11.54 ^a^	0.015 ± 0.0025 ^e^	78,595.6 ± 8955.73 ^a^

Values are given as mean ± standard deviation. Mean with the same Arabic letter in the same column are not significantly different at p˂0.05.

* Kinetic rate determined using linear fit to AA until t_AA50_%. For all fit: R^2^ > 0.86.

** Diffusion coefficient was determined from the analytical solution of the second Fick's law in the case of transient state and for a limited volume of film immersed in a limited volume of solution as proposed by (Crank, 1975). For all fit: R^2^ > 0.86 and RMSE<0.16 mg/L.

An excellent antioxidant activity of 88% was previously shown, similar to the present study (Zhang et al., 2023). The AA of d-Limonene is attributed to its ability to donate a hydrogen atom or electron from its phenolic hydroxyls to stabilize the DPPH radical. Shah and Mehta showed that d-Limonene had a concentration dependent antioxidant activity by reducing the free radical formation in three of four tested assays (DPPH, FRAP and ABTS) except in iron chelating (Shah & Mehta, 2018). As the main principle of this method is the donation of OH-, and as d-limonene lacks the OH- functional groups, the results on the antioxidant activity measured by DPPH method might not completely reflect the real antioxidant potential of this molecule. Therefore, the significantly lower AA values of DL in comparison to CA in this study were attributed to the mentioned effect. While absolute antioxidant activity in foods may differ, DPPH data allow systematic comparison between films with varying polymer composition, essential oil content, and crosslinking. Future studies could complement DPPH with food-relevant assays (e.g., lipid peroxidation or ABTS in emulsions) to confirm the functional performance of released compounds.

The antioxidant potential of sea fennel has attracted a lot of the attention in the past several years (Slišković et al., 2025). Previous studies on Croatian sea fennel extracts have confirmed the good antioxidant activity of the non-volatile fractions (polar/phenolic/aqueous/ethanolic solvent extracts) (Kurek et al., 2024). Literature shows that the antioxidant capacity of the SF was mostly correlated to the content of the chlorogenic acid, generally obtained from liquid extraction using polar or ethanolic solvents (Slišković et al., 2025).

The higher antioxidant activity of CA compared to SF is attributed to the chemical nature, SF being predominantly a d-limonene chemotype. The composition of sea fennel essential oil (same sample used in our study) was confirmed in previous studies using GC–MS analysis, identifying d-limonene as a major component (Kulisic-Bilusic et al., 2010; Šunić et al., 2024b). This reference supports the observed differences in antioxidant activity between CA and SF in the films. Beyond essential oils, this active material has the potential to host a wide range of bioactive molecules and food ingredients with varying polarityes. This versatility could allow the formulation of functional foods enriched with nutraceuticals of interest to the scientific community. For example, gingerols from *Zingiber officinale* have been highlighted as promising bioactive compounds for food applications (Garza-Cadena et al., 2023), mangiferin has demonstrated potential in food systems due to its stability and bioactivity (Castro-Muñoz et al., 2024), and carminic acid can be considered as a natural-based food colorant (Ferreyra-Suarez et al., 2024).

**Fig. 2:**
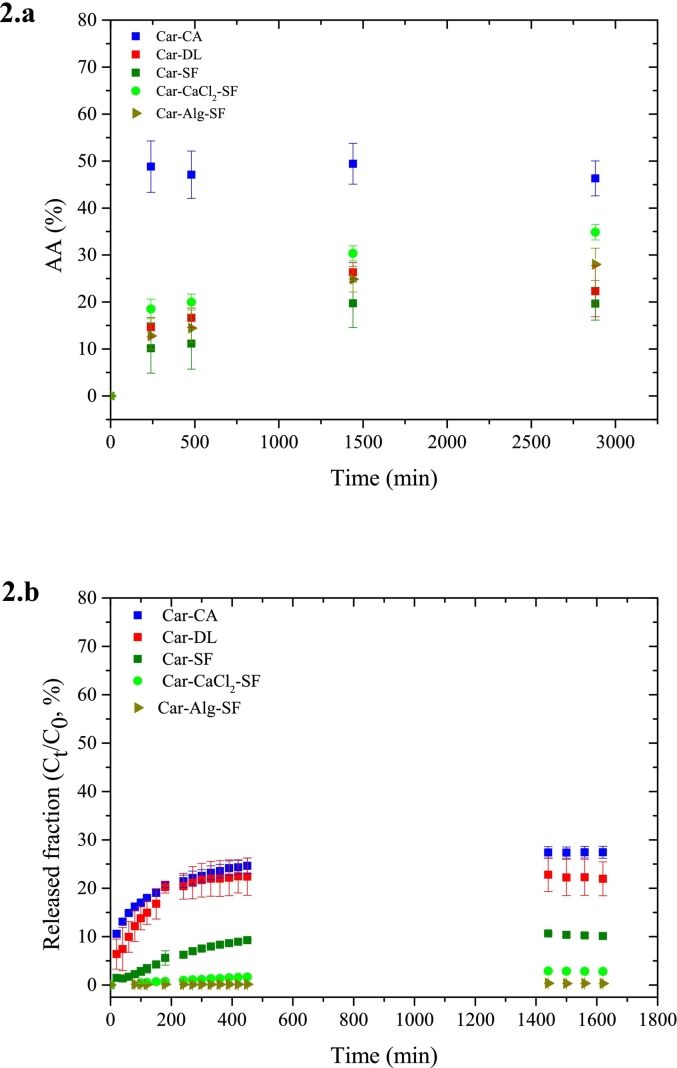
Kinetic of antioxidant activity (AA (%) vs. time) (a) and of release (released fraction (C_t_/C_0_, %) vs. time) (b) of active films (Car-CA, Car-DL, Car-SF, Car-CaCl_2_-SF and Car-Alg). Antioxidant activity and release are measured in ethanol 50% at 25 °C.

The AA of different films is given in Fig. 2a and Table 3. Carrageenan films without active compounds (Car) exhibited an antioxidant activity of 12.79 ± 4.13%. Incorporation of alginate alone (Car-Alg) or sea fennel alone (Car-SF) slightly increased the activity to 14.99 ± 0.77% and 15.81 ± 1.93%, respectively, without statistical significance (*p* < 0.05) (Table 3). It is important to differentiate between statistically significant differences and those that are practically meaningful. Small variations in AA% or release, while statistically detectable, may not translate into substantial functional differences in real-world applications. In contrast, the combined Car-Alg-SF formulation reached a significantly higher activity of 28.03 ± 3.42%, highlighting the cumulative contribution of both sea fennel and alginate. This comparative analysis clarifies that the marked increase in Car-Alg-SF films is not attributable to each component alone. Furthermore, it was previously shown that carrageenan and alginate could have some inherent free radical scavenging, existing due to the possibility of metal ion chelation but still to a limited extent. It was previously shown that the intrinsic antioxidant activity of kappa-Car is attributed to its sulfate groups, which can chelate metal ions and moderately scavenge free radicals (Farhan and Hani, 2017; Prasetyaningrum et al., 2021; Ren et al., 2025). However, its real activity strongly depends on the Car type, whether it is kappa or iota, and therefore results are difficult to compare. In addition, the mechanism of the antioxidant activity of Car and Car-Alg films remains unclear, with, in our knowledge, no data specifically for iota-carrageenan's available in the scientific literature.

The addition of CaCl_2_ into carrageenan network increased the antioxidant activity to reach 26.2% (Table 3) and the mechanism involved remains unclear. The addition of Ca^2+^ to ι-carrageenan was found to induce conformational changes in the carrageenan with the initial coil-to-helix transition. To determine whether CaCl_2_ directly interacts with DPPH free radicals, control experiments were conducted using CaCl_2_ alone. These controls were systematically performed and confirm that the observed trends are reproducible under the experimental conditions. The results show an apparent antioxidant activity of approximately 5% after 48 h, indicating that Ca^2+^ may contribute slightly to DPPH radical scavenging activity (Fig. S1.c). However, this effect remains limited compared with conventional antioxidants. Although Ca^2+^ exhibits a minor but measurable activity, the differences observed in Ca^2+^-crosslinked films are primarily attributed to matrix-mediated effects. In particular, the reduced release of antioxidant compounds from Ca^2+^-crosslinked films is likely associated with the formation of a denser polymer network, which restricts the diffusion of active molecules. Overall, these findings suggest that Ca^2+^ provides a small direct contribution to DPPH scavenging, while a potential synergistic interaction between carrageenan and CaCl_2_ may also contribute to the observed antioxidant behavior.

Some contradictory explanations have been reported in the literature, with no direct activity clearly attributed to CaCl_2_ itself. It is more likely that the observed effect is associated with Ca^2+^ ions rather than the salt as a whole. The presence of Ca^2+^ may induce ionic crosslinking or conformational rearrangements, leading to a more stable structure that could enhance radical scavenging activity (Maletsika et al., 2023; Zhang, Zuo, et al., 2024). Additionally, the increase in ionic strength may influence molecular solvation and potentially affect the DPPH radical system, which could contribute to the observed antioxidant response. These controls were systematically performed and confirm that the observed trends are reproducible under the experimental conditions.

### 3.2 Effect of active compounds properties on both antioxidant activity and release properties of films

Dealing with the active film formulations, the same trend was observed as that of the pure compounds. The highest activity was measured for Car-CA films, with the AA of 46.31%. It took about 22 min to obtain 50% AA (t_AA50%_) with a kinetic scavenging rate equal to 0.55%.min^−1^. On the contrary, Car films with DL and SF showed significantly lower AA ranging from 22.31 to 15.81% for DL and SF, respectively (Table 3). In addition, the kinetic scavenging rate was significantly delayed for both Car-DL and Car-SF films compared to Car-CA films, with the t_AA50%_ being 3 to 4.5 times longer than that observed for CA.

The difference observed in the DPPH kinetics should be related firstly to the difference in the matrix structure, and secondly, to their release kinetics.

The observed release profiles (Figs. 2b and S2) are consistent with Fick's second law, suggesting that diffusion through the polymer matrix is a dominant contribution to mass transfer under the investigated conditions. No pronounced swelling or matrix relaxation effects were detected during the experiments. If present, such effects were likely limited or occurred at time scales that did not significantly alter the overall release behavior. Therefore, the assumption of Fickian diffusion provides a reasonable and practical approximation for describing the release kinetics across all formulations.

For all fittings (Fig. S2 and Table 3), the goodness-of-fit values were R^2^ > 0.86 and RMSE <0.16 mg/L, indicating a satisfactory description of the experimental data. Control films (Car, Car-CaCl₂, Car-Alg) exhibited a rapid initial increase in UV absorbance followed by a plateau (data not shown), which was modeled using a Langmuir-type empirical function for baseline correction, without implying a specific adsorption mechanism.

Although n-values from the Korsmeyer–Peppas model lower than 0.45 are indicative of a Fickian-like release behavior, they should be interpreted as descriptive indicators rather than definitive proof of a purely Fickian mechanism. Possible contributions from polymer relaxation or matrix swelling cannot be entirely excluded, but are expected to remain limited under the controlled experimental conditions used in this study.

From Fig. 2b and Table 3, it can be seen that the fastest release was in CA containing samples (D = 32 ± 3.46 × 10^−15^ m^2^.s^−1^, t_50%_ = 50 min), reaching values of 27.44 ± 1.27%. Compared to CA, the diffusivity from DL (21.95 ± 3.50%) and SF (10.13 ± 0.38%) samples significantly decreased by 4 and by 17 times, respectively.

Although the initial loading of CA in the films was only 1% (*w*/w of biopolymer) compared to 5% for SF, CA from Car-CA films exhibited a higher release percentage (27.44% vs. 10.13% in the SF case). This indicates that the difference in release is primarily due to the higher solubility of CA in the 50% ethanol simulant rather than initial concentration. Since release involves solvent diffusion into the polymer matrix followed by active compound migration, surface hydrophobicity may indirectly affect the early stages of mass transfer. Incorporation of essential oils into alginate-based films has been shown to increase the surface contact angle, making the films more hydrophobic due to migration of oil molecules to the surface during drying (Benavides et al., 2012).

In agreement with recent advances in surface wettability of food packaging materials, the incorporation of active compounds such as essential oils or plant-derived molecules can modify the surface energy and chemical composition of polymeric films, thereby altering their wettability characteristics (Udana Eranda et al., 2025). These modifications may influence interactions with polar solvents and consequently affect diffusion and release mechanisms. Moreover, variations in formulation parameters, such as plasticizer content or the nature of incorporated bioactive compounds, have been reported to significantly impact wettability behavior (Udana Eranda et al., 2025). Thus, it could affect mass transfer phenomena at the film-solvent interface. This modification in wettability can influence interactions with water and polar solvents and consequently may partly contribute to the observed differences in release kinetics. The classification of antioxidant activity as release-controlled or reaction-controlled may vary depending on the medium. In other food simulants, such as aqueous or fatty systems, different solubility and diffusion properties could modify the relative contributions of release and radical-scavenging kinetics. This highlights the importance of considering medium-specific behavior when designing and applying active films.

It should be noted that all experiments were conducted under the same controlled conditions (25 °C) to allow a consistent comparative evaluation of film formulations. Temperature fluctuations encountered in actual food storage, such as refrigeration (∼4 °C) or ambient variations, could influence both release and antioxidant kinetics. Literature reports indicate that higher temperatures generally increase the diffusivity of active compounds from films into food simulants (Benbettaieb et al., 2021). While this study focuses on relative trends, future work is planned to investigate the effect of lower temperatures on release and AA kinetics to better predict the real-world performance of active packaging films. Consequently, the reported kinetic parameters are specific to the experimental conditions (25 °C) used in this study.

Changes in diffusivity can be explained by the partition coefficients. The partition coefficient expresses the ratio of the concentration of the active in the films by the concentration of the active in the food simulants at equilibrium. Measured values showed that the concentration of CA in simulants at the end of the release was significantly (*p* < 0.05) higher than that of DL and SF (Table 3). Consequently, the partition coefficient for DL and SF was from 1.5 to 3.3 times higher than that for the CA. Compared to the antioxidant activity kinetics and the corresponding (AA, t_AA50%_ and AA kinetics rate) parameters, the AA of the active compounds incorporated in the films is closely related to their affinity and solubility in the liquid medium, which is in turn governed by their chemical structure. Chlorogenic acid (CA), for example, is more soluble in ethanol than in water, and thus in solutions with higher ethanol content (as typically used for extraction with ethanol:water ratios of 50:50 to 70:30) (Jeon et al., 2025). Accordingly, CA was released more efficiently in the D1 food simulant (95% ethanolic solution) and in the DPPH medium (DPPH in 50% ethanol) compared to other compounds. On the contrary, as a non-polar terpene, d-limonene exhibits limited solubility in aqueous media, and its solubility decreases significantly as the water content increase. Sea fennel essential oil, like other essential oils, is a complex mixture of hydrophobic volatile compounds (primarily terpenes such as limonene and sabinene), phenylpropanoids, and minor components, and it behaves similarly to other essential oils with respect to solvent polarity. These hydrophobic compounds display slower release and lower diffusion coefficients in polar media due to their poor compatibility with the solvent and interactions with the polymer matrix. This is consistent with literature showing that solvent polarity strongly influences release kinetics, with hydrophobic volatiles exhibiting restricted release and longer times to reach equilibrium compared to hydrophilic antioxidants (Marcuzzo et al., 2012; Nakonechnyi et al., 2025). Therefore, the lower % release, smaller diffusion coefficients, and longer time to reach equilibrium observed for d-limonene and sea fennel essential oil can be mainly explained by their limited solubility in ethanol–water mixtures (≤70% ethanol).

It is important to emphasize that SF is a complex mixture composed of multiple compounds with different physicochemical properties. Therefore, the release kinetics reported here represent a global, phenomenological behavior rather than the individual diffusion of each constituent. Similar approaches have been widely used in studies dealing with essential oil-based films, where the extract is treated as a functional whole and its global release or activity is evaluated (Pranoto et al., 2005). In this context, the present work does not aim to resolve compound-specific release mechanisms, which would require advanced analytical techniques such as GC–MS, but rather to provide a comparative and formulation-level understanding of the release behavior and its impact on antioxidant performance. Chlorogenic acid (CA) and d-limonene (DL) were selected as simplified model compounds to represent contrasted physicochemical behaviors (polar vs. non-polar) relevant to components present in SF. While they do not aim to predict the overall behavior of SF, they provide a useful framework to interpret how molecular properties may contribute to the global release and antioxidant response of the complex extract.

The higher release of chlorogenic acid relative to d-limonene and sea fennel essential oil is closely linked to the polarity of the 50% ethanol simulant, which favors extraction of polar compounds. Therefore, the observed differences in release kinetics are specific to this simulant and should be cautiously extrapolated to real food matrices with different compositions and polarity.

The release profile of sea fennel essential oil was designed to provide an initial burst of antioxidant activity, followed by sustained, slower release. In short shelf-life products such as fresh or minimally processed fish, this approach is advantageous: rapid release quickly mitigates both lipid and protein oxidation during the early critical storage period, while residual activity helps prolong product stability over subsequent days. This qualitative “controlled release” strategy balances immediate protection with extended efficacy. Future studies could establish quantitative target release profiles using kinetic models of lipid and protein oxidation, allowing optimization of antioxidant delivery for specific food matrices and storage conditions.

A quantitative comparison between antioxidant reaction kinetics and release kinetics clarifies the relative timescales of release and antioxidant activity in the films. Antioxidant reaction kinetics indicate how fast the antioxidant (chlorogenic acid, d-limonene, sea fennel extract) react with free radicals (measured by DPPH), whereas release kinetics indicate how fast the antioxidant is released from the active film into the simulant medium. To quantitatively determine the rate (kinetics)-limiting step governing antioxidant performance, a direct comparison between the antioxidant reaction constant (t_AA50%_) and the release kinetics constant (t_50% release_) was performed. Specifically, when t_AA50%_ ≪ t_50% release_, release becomes the rate-limiting step because the antioxidant reacts faster than it is supplied from the matrix. Conversely, when t_50% release_ ≪ t_AA50%_ the chemical reaction governs the overall antioxidant performance.

It is important to distinguish intrinsic antioxidant reactivity from kinetic control. While CA and DL differ in their inherent radical scavenging capacity, the classification of systems as release- or reaction-controlled is based on the relative comparison of t_AA50%_ and t_50% release_, independently of intrinsic reactivity. This comparison provides an empirical and comparative estimate of whether release or intrinsic reaction predominates under the tested conditions. It should be noted that reaching 50% antioxidant activity does not necessarily require 50% compound release; therefore, this approach remains a relative, operational tool rather than a strict mechanistic determination. Although absolute volumes and stirring geometries differ between release and antioxidant assays, the maintained surface-to-volume ratio and sufficient agitation ensure comparable mass transfer conditions, supporting consistent trends across formulations. This comparison is used as an operational, time-based descriptor and not as a strict mechanistic proof of a concentration–response relationship.Within this framework, the concept of “ideal release” reflects a balance between rapid initial antioxidant protection and sustained activity over the product shelf-life, allowing a practical, application-oriented interpretation of release kinetics.

In the case of chlorogenic acid (CA), the antioxidant reaction rate expressed by t_AA50%_ was approximately three times higher than the t_50% release_, quantitatively supporting that release controls the overall antioxidant availability. In contrast, for DL and SF, the closer magnitude of the kinetic constants (t_AA50%_ and t_50% release_) indicates a stronger coupling between diffusion and reaction processes.

In Car-CA films, chlorogenic acid required 50 ± 10 min to reach 50% release (t_50% release_) but only 22 ± 2.6 min to reach 50% of its maximum antioxidant activity (t_AA50%_) (Table 3). This indicates that the antioxidant reaction occurs faster than the release, likely due to the high radical-scavenging capacity of chlorogenic acid, which is related to its meta- and para-hydroxyl groups on the phenolic ring. In this case, the release was not the limiting step for antioxidant activity in the Car-CA films. In contrast, d-limonene exhibited a t_50% release_ of 87.5 ± 3.5 min and t_AA50%_ of 70 ± 14 min (Table 3), indicating that the antioxidant activity was more dependent on the release rate. Similar behavior was observed for sea fennel essential oil. To our knowledge, systematic studies directly comparing release kinetics and antioxidant reaction kinetics for active compounds incorporated into packaging films are still scarce, highlighting the novelty of the present kinetic-based approach. This approach provides comparative insight under identical conditions rather than a full mechanistic description of coupled processes.

**Fig. 3:**
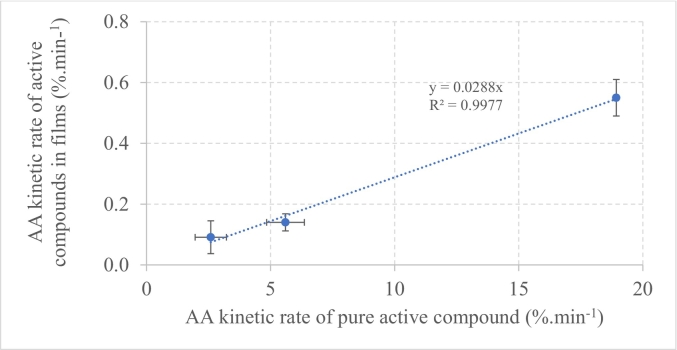
Antioxidant reaction rate of the active compounds incorporated in film as a function of their rate when introduce as pure compound in the DPPH solution.

The correlation between kinetic rate of the antioxidant reaction of the pure active compounds in DPPH solution and that of same compounds incorporated in films is plotted in Fig. 3. The kinetic rate is reduced by about 35 times (1/slope) when the active compounds are incorporated into film matrices. Even though only 3 compounds have been tested, the linear correlation is significant between intrinsic activity of pure CA, DL or SF and their activity when encapsulated in carrageenan-based films. This demonstrated that films strongly delay/reduces the antioxidant power of these compounds.

Such concentration–activity relationships have previously been reported. Iñiguez-Franco et al. showing positive correlations between antioxidant concentration and percentage of DPPH radical scavenging for catechin (R^2^ = 0.99) and epicatechin (R^2^ = 0.91) (Iñiguez-Franco et al., 2012). Similar behavior was observed during migration studies of catechin, epicatechin, quercetin, gallic acid, caffeine, and other phenolic compounds into food simulants, where antioxidant activity was directly proportional to antioxidant concentration in the simulant (López-de-Dicastillo et al., 2010; Ortiz-Vazquez et al., 2011). Likewise, in extracts from different grape varieties, antioxidant activity increased with phenolic compound concentration. These findings reinforce that the effective antioxidant response is strongly dependent on the amount of compound available in the reaction medium.

**Fig. 4:**
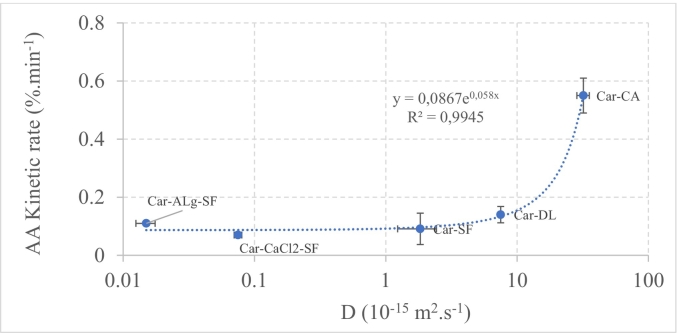
Relation between the release diffusion coefficient of active compounds incorporated into carrageenan-based films and their antioxidant activity.

However, considering the release rate impact on the antioxidant activity of films, a clear influence of the diffusivity is displayed in Fig. 4. An exponential dependence of the DPPH activity on the diffusivity of the active compounds released from the biopolymer matrix is significantly demonstrated. This means delayed release induced slower antioxidant activity. Consequently, the main limiting factor is clearly established as being the release rate. This interpretation applies within the experimental framework and should be considered at the formulation level. Indeed, even if intrinsic activity is quite low, its rate remained always much faster than when incorporated in film. This behavior is consistent with previous findings reported in antioxidant bilayers based on PHBV and plasticized electrospun PLA-PHB fibers encapsulating catechin (Arrieta et al., 2019). The antioxidant activity over time closely followed the catechin release profile, confirming that radical scavenging efficiency is strongly governed by release kinetics.

Controlled or sustained release systems (e.g., encapsulation, active packaging) aim at delivering bioactive antioxidants at a rate that preserves their functional activity over time. Gulcin displayed that the antioxidant activity is determined not only by molecular structure but also by kinetics of reaction mechanisms that govern radical scavenging efficiency and is dependent on the availability of the compound (Gulcin, 2025). Even if a compound has inherently high antioxidant potential, inadequate release kinetics can limit its effective in situ concentration, preventing full exploitation of its structure-dependent activity (Chen et al., 2015).

### 3.3 The effect of polymer interactions and crosslinkers on the kinetics of antioxidant activity and release

The effect of both biopolymer-biopolymer interaction (Car-Alg) and of the bivalent cation Ca^2+^ (Car-CaCl_2_) on the AA and the release kinetics of SF was also studied. The released content of SF from Car films (10.13 ± 0.38%) decreased by 72% and by 96% when CaCl_2_ or Alg were, respectively, added to carrageenan (Table 3).

The time needed to release 50% of SF was increased by more than 2 times after the addition of both Alg or Ca^2+^ (Table 3 and Fig. 2b). Accordingly, the diffusion coefficient of SF was also significantly decreased by 95% and by 99% (about 100 times) when CaCl_2_ or Alg were respectively added into Car-SF films. Different effects of CaCl2 or Alg may be related to stronger intermolecular interactions and a more constrained polymer organization in the presence of Ca^2+^, which could contribute to the delayed release of SF. Iota carrageenan contains two negatively charged sulfate groups (-OSO_3_^−^) that react with the Ca^2+^ ions to form ionic cross-links. This more constrained organization may reduce molecular mobility and consequently slow down SF diffusion and release. Therefore, it can be concluded that calcium ions play a pivotal role in modulating the release of active compounds from active films by influencing the film's structural integrity and permeability.

Direct measurements of mesh size or crosslink density were not performed in this study. However, slower release kinetics observed here are consistent with our recent work (Avidos et al., 2026) where similar formulations showed increased crosslinking and denser polymer networks using structural characterization techniques. Therefore, the structural interpretation proposed here should be considered as a formulation-level hypothesis consistent with the observed release behavior and previous literature, rather than a direct structural proof obtained in the present study.

In the case of mixing Car with Alg, the most probable explanation is the formation of interpenetrating biopolymer network (IPN). In these structures, both biopolymers retain their individual structures but are physically entangled simultaneously leading to enhanced barrier properties. Indeed, it was previously shown that Car-Alg blend formed a more ordered or constrained structure through intermolecular interactions, with hydrogen bonds or ionic association between their functional groups (Adão et al., 2024).

These large variations on the release kinetics parameters after Alg or Ca^2+^ addition, reflect the strong influence of crosslinking and matrix interactions on molecular mobility within the films. Although the magnitude of these changes may be higher than what is typically expected under practical food packaging scenarios, they provide useful mechanistic insight into how film composition can control release behavior.

While the presence of crosslinker (Ca^2+^) significantly increased (*p* < 0.05) the antioxidant activity at equilibrium of Car-SF films, the kinetic was shown to be different: more time was needed to reach 50% of activity (t_AA50%_ rises) and the kinetic rate of AA decreased. The presence of Ca^2+^ could result in some electrochemical reaction between Ca^2+^ and DPPH free radical which increased the AA of crosslinked Car-CaCl_2_-SF films as previously shown.

In the case of Alg, its addition increased the AA at equilibrium. This can be explained by the natural AA of the sodium alginate (Ning et al., 2022). Briefly, the antioxidant activity of alginate is likely attributed to the radical scavenging primarily via hydrogen atom transfer that is related to the α (1–4) glycosidic linkage in the G blocks whereas increases in the G blocks flexibility influences the availability of sodium alginate OH-groups and the ability to donate H-atom (Sari-Chmayssem et al., 2016). In carbohydrates, electron transfer is less common, while hydrogen atom transfer is the predominant antioxidant mechanism. Radical addition is generally unlikely due to the absence of double bonds or aromatic rings in most carbohydrate structures. Therefore, the observed increase in antioxidant activity over treatment time may be associated with the formation of double bonds between the C-4 and C-5 positions, which could enhance radical stabilization (Kelishomi et al., 2016). From both kinetics (AA and release), the addition of the crosslinker or of the Alg into Car-SF films delayed the release of SF in 50% ethanolic food simulant (% of release and diffusivity decreased) but increased the AA. The effect of Ca^2+^ or Alg on the release (delay) is a dynamic and physical effect on the biopolymers network (entrapped of SF), whereas it has opposite effect on the antioxidant activity, probably because of a chemical reaction (reaction with the DPPH free radicals). The AA of SF in Car-Alg or Car-CaCl_2_ films was not related to the % of release and thus the independent bioactivity-release relationship was observed. It is essential to mention that a rapid release of the active agent is not desirable since it may promote the diffusion to internal parts of the food, reducing the protection action at the surface. Alternatively, if the release rate is very slow, its AA effect cannot be reached. Hence, the knowledge of the release rate and, therefore, the diffusivity of the active agents from the film matrix is a determinant factor in the development of active films (Bierhalz et al., 2014).

Future research should also focus on the long-term stability of the developed active films during storage. In particular, evaluating their physicochemical, mechanical, antioxidant, and release properties under varying environmental conditions (temperature, relative humidity, salt content, light exposure, and pH) prior to application is a key next step to ensure practical applicability of these materials. A QSAR model could theoretically help explain why chlorogenic acid (CA) reacts faster than d-limonene (DL) or sea fennel oil (SF) by linking their molecular structures to observed release and antioxidant kinetics and therefore provide mechanistic insight into structure-activity relationships. However, due to the limited number of compounds studied and the influence of polymer matrix effects on release and reactivity, a statistically meaningful QSAR analysis is not feasible in this work. Instead, compound-specific behaviors were interpreted qualitatively and quantitatively based on known physicochemical properties, with QSAR approaches proposed as a perspective for future studies.

## 4 Conclusions

This study demonstrates the potential of carrageenan-based films enriched with natural bioactives (chlorogenic acid (CA), d-limonene (DL), and sea fennel essential oil (SF)) for antioxidant-active food packaging. By coupling release kinetics with antioxidant reaction kinetics, a mechanistic understanding was established of how molecular structure governs functional performance. A strong linear correlation between the intrinsic antioxidant activity of the pure compounds and their activity once incorporated into films was observed, although the reaction rate was reduced by approximately 35-fold in the film systems. This clearly confirms that the release rate constitutes the main limiting factor controlling antioxidant effectiveness in the polymer matrix.

Antioxidant performance was compound-specific. CA exhibited rapid and pronounced activity due to its polarity and high solubility, with release identified as the rate-determining step. In contrast, DL and SF showed slower diffusion and reduced activity, primarily due to matrix constraints and lower solubility. Structural modulation via alginate incorporation and Ca^2+^ crosslinking decreased diffusivity and prolonged release. In SF-containing films, network modifications enhanced antioxidant effects, suggesting that additional matrix-related interactions may contribute beyond simple diffusion control.

The present results indicate that CA provides rapid antioxidant protection, whereas DL and SF contribute more gradually. Although combining these compounds could hypothetically optimize both immediate and sustained antioxidant effects, this remains a hypothesis for future investigation rather than an experimentally demonstrated result. Similarly, applying quantitative structure–activity relationship (QSAR) approaches to such complex, matrix-dependent systems is highly challenging, as interactions between multiple compounds and the polymer network can significantly influence release and activity, requiring careful experimental validation.

The novelty of this work lies in the systematic, comparative analysis of release kinetics and antioxidant activity across multiple film formulations, using both well-defined model compounds and a complex extract. While the correlations presented do not constitute a predictive kinetic model, they provide an experimentally grounded framework to compare and rank formulations based on their release–activity profiles, guiding formulation-level design decisions without over-interpreting the mechanistic behavior of complex mixtures. It should be noted that establishing a direct, quantitative link between specific structural properties (e.g., mesh size, crosslink density) and transport parameters (e.g., diffusion coefficients) was beyond the scope of this study; instead, the trends observed across different formulations provide comparative mechanistic insight and inform future, more detailed investigations.

Furthermore, DPPH radical scavenging reflects only one dimension of antioxidant potential and does not encompass the full complexity of oxidative processes occurring in real food systems. This concept remains speculative and therefore, the results should be interpreted with caution when extrapolating to food systems.

Overall, this kinetic-driven approach underscores the necessity of integrating intrinsic reaction kinetics with mass transport phenomena to rationally design active packaging systems. The proposed framework provides a basis for tailoring release profiles according to targeted shelf-life requirements. Future work should validate these findings in real food matrices, assess long-term stability during storage, and investigate interactions with food components to support the development of sustainable and responsive packaging materials.

## CRediT authorship contribution statement

**Lilou Avidos:** Writing – original draft, Methodology, Investigation, Formal analysis. **Petra Pišonić:** Methodology, Formal analysis, Data curation, Conceptualization. **Mia Kurek:** Writing – review & editing, Visualization, Project administration, Methodology, Investigation, Funding acquisition, Formal analysis, Data curation, Conceptualization. **Frédéric Debeaufort:** Writing – review & editing, Visualization, Validation, Supervision, Resources, Project administration, Methodology, Investigation, Funding acquisition, Formal analysis, Data curation, Conceptualization. **Nasreddine Benbettaieb:** Writing – review & editing, Writing – original draft, Visualization, Validation, Supervision, Software, Resources, Project administration, Methodology, Investigation, Funding acquisition, Formal analysis, Data curation, Conceptualization.

## Declaration of competing interest

The authors declare that they have no known competing financial interests or personal relationships that could have appeared to influence the work reported in this paper.

## Data Availability

No data was used for the research described in the article.

## Appendix A Supplementary data

Supplementary material **Figure S1.** Time-dependent change in optical density at 517 nm of DPPH solution (50mg.L^-1^ in ethanol/water, 50:50 v/v) without **(a)** and with CaCl_2_ (0.8mg/10mL of DPPH) **(b)**, and corresponding antioxidant activity (AA, %) of CaCl_2_ over time (min) **(c)**. **Figure S2.** Release kinetics of CA (a), DL (b) and SF (c) from Carrageenan films (Car) into D1 food simulant (ethanol 50% at 25°C). Release kinetics of SF from (c) Carrageenan films (Car), (d) Carrageenan-CaCl_2_ films and (e) Carrageenan-Alginate films (Car-Alg) into D1 food simulant (ethanol 50% at 25°C). (C_t_, concentration of active released in the food simulant at time t; C_∞_ concentration of active released in the food simulant at equilibrium. The lines are the fitting of the experimental values (symbols) from release kinetics data).
