# Supplementary material for: Kinetical approach of release and antioxidant performances of ι-carrageenan-based films modified by sodium alginate and Ca2+ and incorporating sea fennel essential oil and its major components
Source: Food Chem X. 2026 Jun 30;37:104155. doi: 10.1016/j.fochx.2026.104155 (PMC13355736; doi:10.1016/j.fochx.2026.104155)
Supplement: Supplementary material — Figure S1. Time-dependent change in optical density at 517 nm of DPPH solution (50mg.L-1 in ethanol/water, 50:50 v/v) without (a) and with CaCl2 (0.8mg/10mL of DPPH) (b), and corresponding antioxidant activity (AA, %) of CaCl2 over time (min) (c). Figure S2. Release kinetics of CA (a), DL (b) and SF (c) from Carrageenan films (Car) into D1 food simulant (ethanol 50% at 25°C). Release kinetics of SF from (c) Carrageenan films (Car), (d) Carrageenan-CaCl2 films and (e) Carrageenan-Alginate films (Car-Alg) into D1 food simulant (ethanol 50% at 25°C). (Ct, concentration of active released in the food simulant at time t; C∞ concentration of active released in the food simulant at equilibrium. The lines are the fitting of the experimental values (symbols) from release kinetics data). [file mmc1.docx]

**Figure S1.** Time-dependent change in optical density at 517 nm of DPPH solution (50mg.L^-1^ in ethanol/water, 50:50 v/v) without **(a)** and with CaCl_2_ (0.8mg/10mL of DPPH) **(b)**, and corresponding antioxidant activity (AA, %) of CaCl_2_ over time (min) **(c)**.

**Figure S2.** Release kinetics of CA (a), DL (b) and SF (c) from Carrageenan films (Car) into D1 food simulant (ethanol 50% at 25°C). Release kinetics of SF from (c) Carrageenan films (Car), (d) Carrageenan-CaCl_2_ films and (e) Carrageenan-Alginate films (Car-Alg) into D1 food simulant (ethanol 50% at 25°C). (C_t_, concentration of active released in the food simulant at time t; C_∞_concentration of active released in the food simulant at equilibrium. The lines are the fitting of the experimental values (symbols) from release kinetics data).
